# Introducing the Event Related Fixed Interval Area (ERFIA) Multilevel Technique: a Method to Analyze the Complete Epoch of Event-Related Potentials at Single Trial Level 

**DOI:** 10.1371/journal.pone.0079905

**Published:** 2013-11-04

**Authors:** Catherine J. Vossen, Helen G. M. Vossen, Marco A. E. Marcus, Jim van Os, Richel Lousberg

**Affiliations:** 1 Department of Anesthesiology and Pain Medicine, Maastricht University Medical Centre, The Netherlands; 2 Amsterdam School of Communication Research, University of Amsterdam, Amsterdam; 3 Department of Psychiatry & Psychology, Maastricht University, The Netherlands; 4 King’s College London, King’s Health Partners, Department of Psychosis Studies, Institute of Psychiatry, London,; Université catholique de Louvain, Belgium

## Abstract

In analyzing time-locked event-related potentials (ERPs), many studies have focused on specific peaks and their differences between experimental conditions. In theory, each latency point after a stimulus contains potentially meaningful information, regardless of whether it is peak-related. Based on this assumption, we introduce a new concept which allows for flexible investigation of the whole epoch and does not primarily focus on peaks and their corresponding latencies. For each trial, the entire epoch is partitioned into event-related fixed-interval areas under the curve (ERFIAs). These ERFIAs, obtained at single trial level, act as dependent variables in a multilevel random regression analysis. The ERFIA multilevel method was tested in an existing ERP dataset of 85 healthy subjects, who underwent a rating paradigm of 150 painful and non-painful somatosensory electrical stimuli. We modeled the variability of each consecutive ERFIA with a set of predictor variables among which were stimulus intensity and stimulus number. Furthermore, we corrected for latency variations of the P2 (260 ms). With respect to known relationships between stimulus intensity, habituation, and pain-related somatosensory ERP, the ERFIA method generated highly comparable results to those of commonly used methods. Notably, effects on stimulus intensity and habituation were also observed in non-peak-related latency ranges. Further, cortical processing of actual stimulus intensity depended on the intensity of the previous stimulus, which may reflect pain-memory processing. In conclusion, the ERFIA multilevel method is a promising tool that can be used to study event-related cortical processing.

## Introduction

In psychophysiological pain research, the event-related potential (ERP), a time-locked derivative of the electroencephalogram (EEG), is frequently used as an objective measure of pain [1,2]. Since 1970, many reports have investigated the pain ERP. In particular, the N2 and N2-P2 peak-to-peak amplitude in the pain ERP are associated with stimulus characteristics, such as intensity [1,3,4,5,6], and processes, such as attention [7,8] and habituation [9,10,11,12,13].

To identify and examine these peaks in the ERP, several methods have been developed, of which domain averaging across trials and conditions has been the most commonly used in the past [14,15]. In this type of analysis, the continuous EEG signal is partitioned into stimulus-related time segments (epochs). Invalid epochs, confounded by artifacts, such as eye blinks, are identified and removed or corrected [16].

Next, an averaging procedure of all valid epochs is performed intraindividually (all epochs of a single person) and interindividually (all epochs of a group or experimental condition), resulting in averaged ERPs. On visual inspection of an averaged ERP, several positive and negative peak amplitudes can be identified in the post-stimulus period. Around these averaged peak amplitudes, time windows are defined to determine the maximum (or minimum) amplitude of the peaks per subject or condition. In addition, latencies (time after stimulus onset) of these peak values are determined. These stimulus-related peak and latency values serve as dependent variables in statistical analyses, such as ANOVA [17]. There are, however, certain disadvantages of this procedure. First, the method of averaging assumes that the ERP waveform is stable over time with respect to amplitude and latency. Consequently, by averaging, across-trial variability of the ERP is lost [18,19]. Across-trial variability in amplitude, however, may be important—for example, in the study of habituation to repeated stimuli. Another problem is the trial-to-trial variability in latency, so-called latency jitter [20]. In the worst case, when latencies of these peaks vary considerably across trials, specific peaks may be undetectable after averaging (for an extensive review, see 18). Further, when using ANOVA as a statistical technique to analyze ERP data, subjects are deleted list-wise when one or more missing values occur. However, missing data—for example, due to EOG artifacts—are common in ERP analysis, which can lead to a considerable loss of analyzable cases.

In recent years, several advances have been made in signal processing and statistical analysis of ERPs. Methods have been developed to enhance the signal-to-noise ratio and reduce latency jitter, such as the use of continuous wavelet transform (CWT) [18,21,22] and independent component analysis (ICA) [23]. Furthermore, automatic single-trial measurements of the filtered waveforms, using a multiple linear regression have been developed, in which peaks of single trials are estimated and derived from the parameters of the across-trial filtered waveform [6,21,24,25].

With respect to the statistical analysis of ERPs, the introduction of multilevel random regression analysis has been proposed. A recent ERP study by Vossen and colleagues demonstrated the superiority of multilevel analysis over repeated measures ANOVA [26]. Unlike ANOVA, multilevel analysis takes into account the hierarchical structure of ERP data, in which trials are nested within subject [26,27]. In addition, in multilevel analysis, all valid EOG artifact-free trials can be included, and cases are not deleted list-wise. Finally, random effects and nonlinear contrasts can be incorporated. Thus, person-by-time effects, such as habituation and its nonlinear properties, can be modeled [26]. 

Altogether, many advances in the analysis of event-related potentials have been made. However, the main focus was always on peaks and their latencies. Undoubtedly, (maximized) peak values carry relevant information on various processes [1,7,28,29]. Theoretically, however, *each* post-stimulus point on the waveform contains potentially meaningful information, regardless of whether it is peak-related. Ideally, one would want to explain the entire variability in amplitudes at each latency point after a stimulus using a series of variables that modify the amplitude.

Stated mathematically, the variability of amplitudes on a specific post-stimulus latency point is a function of stimulus-related variables and variables that pertain to all other ongoing brain processes. From this perspective, EEG data should be analyzed on a single-trial level and as ‘raw’ and untransformed as possible. The area under the curve measure (AUC) may be useful for avoiding an excessive amount of analyses (there are an infinite number of latency points) [30]. In ERP research, the AUC method has not been applied often, and when it has been used, it is related to areas around peaks [31].

**Figure 1:**
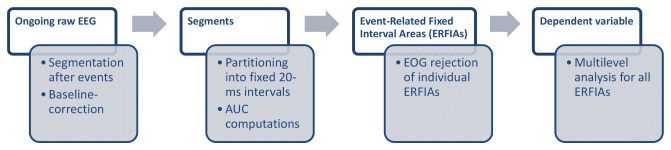
The ERFIA multilevel method. The processing steps from the ‘raw’ EEG to ERFIAs serving as the dependent variable for multilevel analysis. First, the EEG is partitioned into event-related segments, and then a baseline correction is made. Next, the segments are partitioned in 20-ms intervals, and the area under the curve for every interval for all trials is calculated. As a third step, an EOG rejection is carried out for all ERFIAs separately. Finally, the valid ERFIAs per fixed interval serve as a dependent variable in the multilevel analysis.

Based on these considerations, this article presents an alternative method of analyzing event-related EEG data, which focuses on post-stimulus fixed-interval areas, independent of peaks, wherein the concept of AUC is applied at single-trial level and analyzed by multilevel regression analysis. In practice, for each single trial, the post-stimulus EEG information is partitioned into small fixed-interval AUC segments (See Figure 1). These event-related fixed-interval AUCs (ERFIAs) are nested within subjects and should, therefore, be analyzed using a multilevel regression technique—ie, we attempt to explain the variance in event-related EEGs for every fixed-area post-stimulus on 2 levels: between subjects and within subject. Pilot analyses have suggested that this method is productive [32].

Based on the results of the pilot study, we hypothesized that the ERFIA multilevel method generates results that are comparable with those of (multilevel) peak analyses. Specifically, we expect that ERFIAs in the N2-P2 region correlate significantly with stimulus intensity. To test this hypothesis, we reanalyzed an existing ERP dataset of a (non-)noxious stimulus rating paradigm [26]. In addition, the effects of stimulus intensity, previous stimulus intensity, and (nonlinear) habituation were explored in non-peak-related areas of the pain-related somatosensory ERP up to 1500 ms post-stimulus. 

## Materials and Method

### Ethics Statement

Approval was obtained from the medical ethics committee of the Academic Hospital Maastricht, on January, 6th, 2005. All subjects gave their verbal and written informed consent prior to the study, after having read a document with detailed information of the study and having discussed any possible concerns with the researcher.

### Subjects

Eighty-five pain-free subjects participated in the study, ranging in age from 18 to 65 years. Exclusion criteria were a history of chronic pain complaints, the use of psychoactive drugs and the use of analgesics less than 8 hours prior to the experiment. Participation was rewarded with twenty-five € on completion of the study. 

### Stimuli

Electrical pulse stimuli (duration 10 milliseconds) were applied intracutaneously on the left middle finger, per Bromm and Meier [3]. Using this method, a small lumen in the epidermis was prepared, using a dental gimlet, ensuring that the procedure was not painful. In the prepared lumen, a golden electrode was placed and fixed with tape. Two grounding copper laces were attached around the prepared finger and wrist. First, the sensation and pain thresholds were determined by gradually increasing the intensity of the stimulus, starting at zero intensity. The first intensity that was consciously experienced was defined as the sensation threshold; the first intensity that was experienced as painful was defined as the pain threshold. This procedure was repeated 3 times to generate a reliable measurement. Based on the difference between a subject’s sensation and pain thresholds, 5 stimulus intensities were presented in a rating paradigm. One of the 5 intensities was equal to the pain threshold, against which the other intensities were defined: -50%, -25%, +25%, and +50% of the difference between the sensation and pain thresholds (threshold range). The maximum stimulus intensity never exceeded 5 mA.

### Paradigm

One hundred fifty stimuli were presented in a rating paradigm [3]. The 5 stimulus intensities were presented semi-randomly. Blocks of 15 stimuli were administered, in which each intensity occurred 3 times. Inter-stimulus intervals (ISIs) ranged between 9 and 11 seconds. Subjects were asked to rate the intensity of each stimulus on a scale from 0 (no sensation) to 100 (the most excruciating pain imaginable). 

### EEG recording

All EEG recordings were conducted in an electrically- and sound-shielded cubicle (3*4 m^2^). Ag/AgCl electrodes were placed on Fz, Cz, Pz, C3, C4, T3, and T4 using the international 10-20 system [33]. Impedances were maintained below 5 kΩ. A reference electrode was placed on each ear lobe. To check for possible vertical eye movements, an electrooculogram (EOG) electrode was placed 1 centimeter under the midline of the right eye. A ground electrode was placed at Fpz. All electrodes were fixed using 10-20 conductive paste. Neuroscan 4.3 software was used to record EEGs.

### Procedure

Before the start of the experiment, the subjects were informed about the purpose of the study. Subjects were told that they would undergo EEG registration while receiving various intensities of electric shocks—some painless, some painful. After informed consent forms were signed, EEG electrodes were attached, and the shock electrode was placed on the top of the left middle finger as described by Bromm and Meier [3]. Next, the sensation and pain thresholds were determined, after which the rating paradigm was initiated. 

### Data reduction and computation of ERFIAs

EEGs were recorded at a 1000-Hz sampling rate using Neuroscan 4.3. Trials were segmented from the continuous EEG, from 200 ms before the stimulus to 1500 ms post-stimulus. Data were offline band-pass filtered (0-50Hz) and baseline-corrected (interval -200 ms to 0 ms) using BrainVision Analyser 2.0, Brain Products, München, Germany. The filtered data segments were exported to Microsoft Office Excel 2007. Twenty-millisecond ERFIAs were calculated from 0 to 1500 ms post-stimulus, resulting in 75 ERFIAs per trial per EEG electrode per subject. Additionally, maximum and minimum values of the EOG channel were selected per 20-ms ERFIA. Next, the ERFIAs and maximum and minimum EOG values of all 7 electrodes were imported into SPSS 18.0. Single ERFIAs with EOG activity that exceeded ±25µV were excluded from the multilevel analyses. The number of rejected ERFIAs ranged from 5.6% to 24%, depending on ERFIA interval and location. After EOG rejection, a minimal amount of 8600 ERFIAs was available for multilevel analysis. 

### Statistical analyses

**Figure 2:**
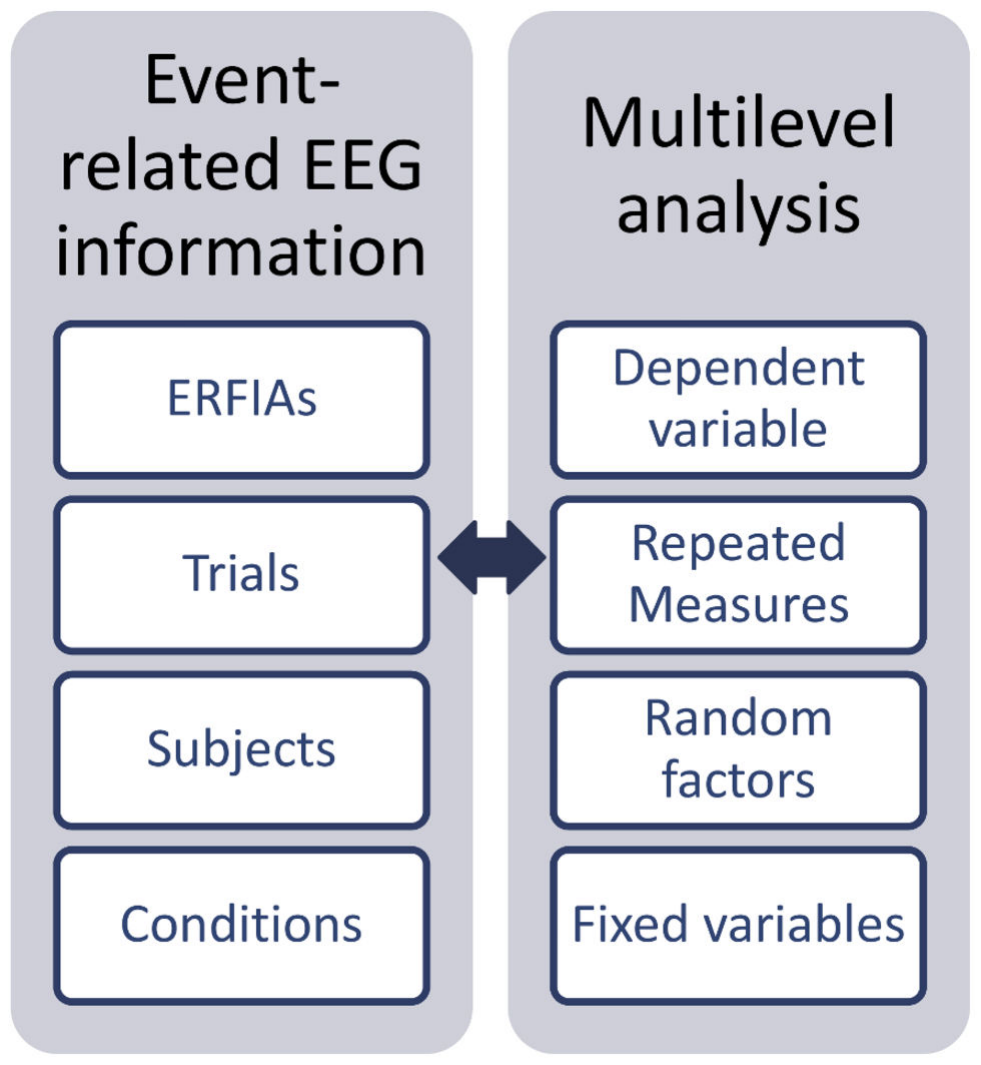
Multilevel analyses for event-related EEG. Valid ERFIAs for each fixed interval serve as the dependent variable. Trials are nested within subjects and are selected as repeated measures. Subjects represent the highest level in the model, and random factors can be modeled, such as stimulus intensity and trial number. Conditions can be incorporated as fixed factors in the model.

Multilevel random regression analyses were carried out separately for each EEG electrode. Trial number (1-150 stimuli) was considered the repeated measure. Subjects represented the highest level in the model, and the 20-ms ERFIAs were the dependent variable (see Figure 2). As shown in Figure 1, ERFIAs were derived from single trials and were computed independently of averaged waveforms.

One of the advantages of multilevel analysis is that it models time or trial effects with a nonlinear function of trial number. As in the study by Vossen and colleagues, habituation was modeled by 3 time effects [26]. First, a linear effect was modeled, assuming a linear decrease or increase of the dependent variable (of a particular ERFIA) over time. Second, an inverse relationship was included, representing a rapid decline in the consecutive ERFIAs, followed by a gradual decline or plateau phase—ie, habituation of the initial trials is more pronounced than that later in the experiment. Third, a quadratic function, representing a sensitization process (or dishabituation) after an initial habituation, was modeled [34]. Thus, habituation was modeled in three ways: linear habituation (trial number), fast habituation (inverse relationship, computed as 1/trial), and dishabituation (parabolic relationship, computed as trial*trial) [26]. 

The full multilevel model comprised the following independent variables (fixed factors): actual stimulus intensity, previous stimulus intensity, the interaction between actual stimulus intensity and previous stimulus intensity, trial, trial_inverse_, trial_quadratic_, age, gender, and absolute stimulus intensity level of the difference between sensation threshold and pain threshold. We made the assumption that subjects differ from each other in their response to the 5 intensities and with regard to habituation. Thus, random effects, such as a random intercept and a random slope for intensity and (linear) trial number, were also included. The Scaled Identity covariance structure was used in the multilevel analyses.

The analyses were performed separately for each 20-ms ERFIA for all 7 cranial sites, resulting in 75 (1500 ms/20 ms) * 7 (cranial locations) = 525 multilevel models. For this large number of statistical tests, a correction for multiple testing should be performed. We chose not to define a specific *P*-value for statistical significance, due to the partially explorative aspect of the analyses. Instead, we considered relatively long-lasting effects (3 or more consecutive 20-ms ERFIAs) with P-values <= 0.05 as significant.) Single ERFIAs were considered significant when the P-value exceeded 0.0007 (with a corresponding T-value of 3.43), based on Bonferroni correction for the complete epoch, obtained by dividing a significance level of 0.05 by the number of ERFIAs (74). The full multilevel model is described in the appendix. All statistical analyses were performed with SPSS 18.0. 

## Results

### Subject characteristics

Eighty-five subjects participated in the study; 9 were excluded because they had significant pain in the previous week or had consumed more than 5 units of alcohol on the evening before the experiment. Ultimately, there were 76 analyzable cases-26 men (34.2%) and 50 women (65.8%). The mean age of the participants was 34.8 years (SD = 13.7). 

### Grand averaged EEG response for 5 stimulus intensities and habituation

**Figure 3:**
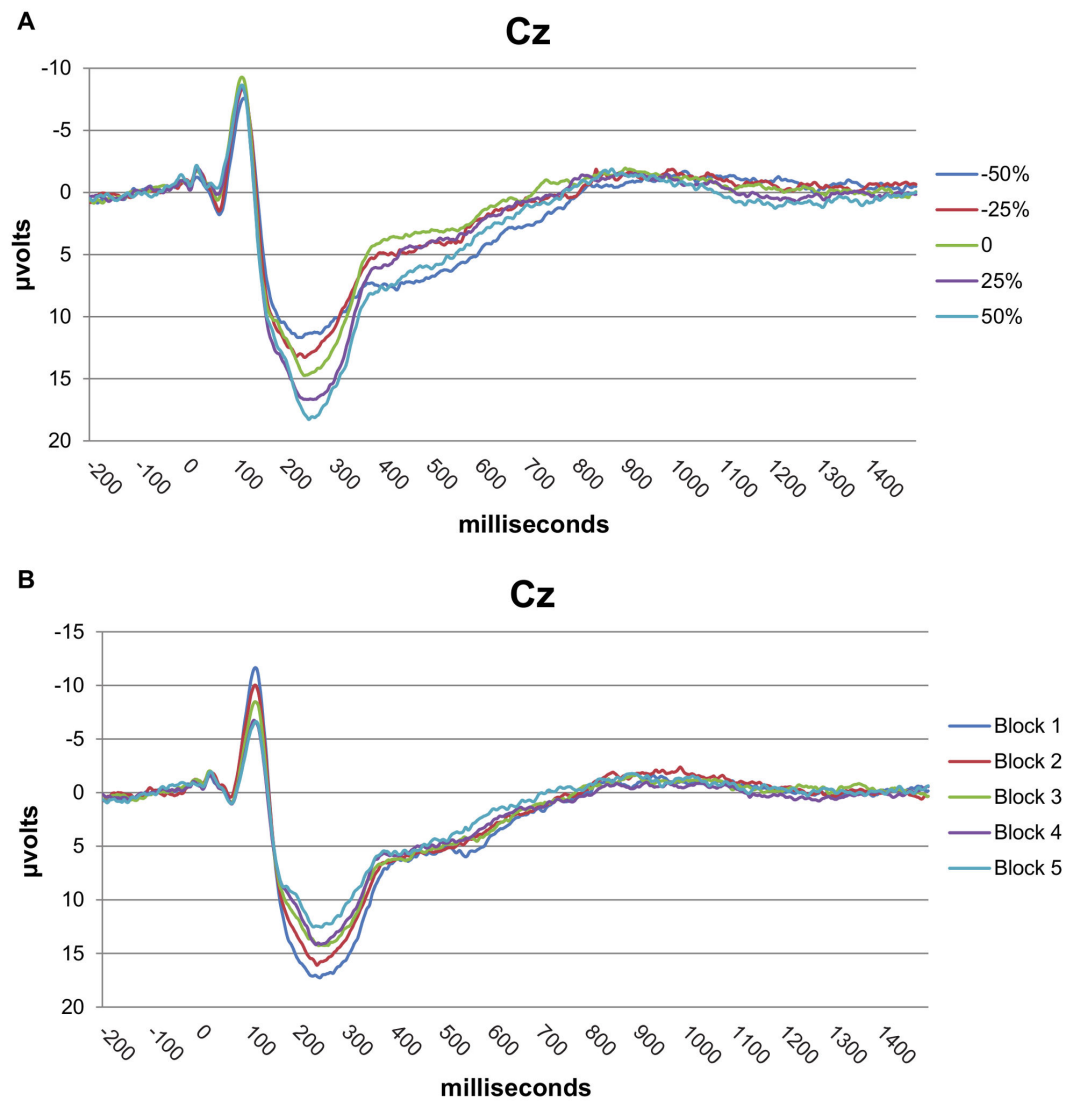
Grand averages of intensity and habituation. A) Grand average for 5 stimulus intensities. B) Grand averages for 5 consecutive blocks of 30 stimuli. Both peak- and non-peak related EEG information can differ between the conditions. Note that these averages are only for illustrative purposes and were not used for the computation of event-related fixed interval areas (ERFIAs) at single trial level.

In Figure 3, the grand averages are displayed for the 5 stimulus intensities and linear habituation at Cz. Notably, intensity and habituation affected not only the N2 and P2 peaks but also their slopes and nonpeak-related latencies. (Note that these graphs are for illustrative purposes only—the averages were not used in the ERFIA multilevel analyses).

### Interpretation of the model parameters from the ERFIA multilevel analyses

**Figure 4:**
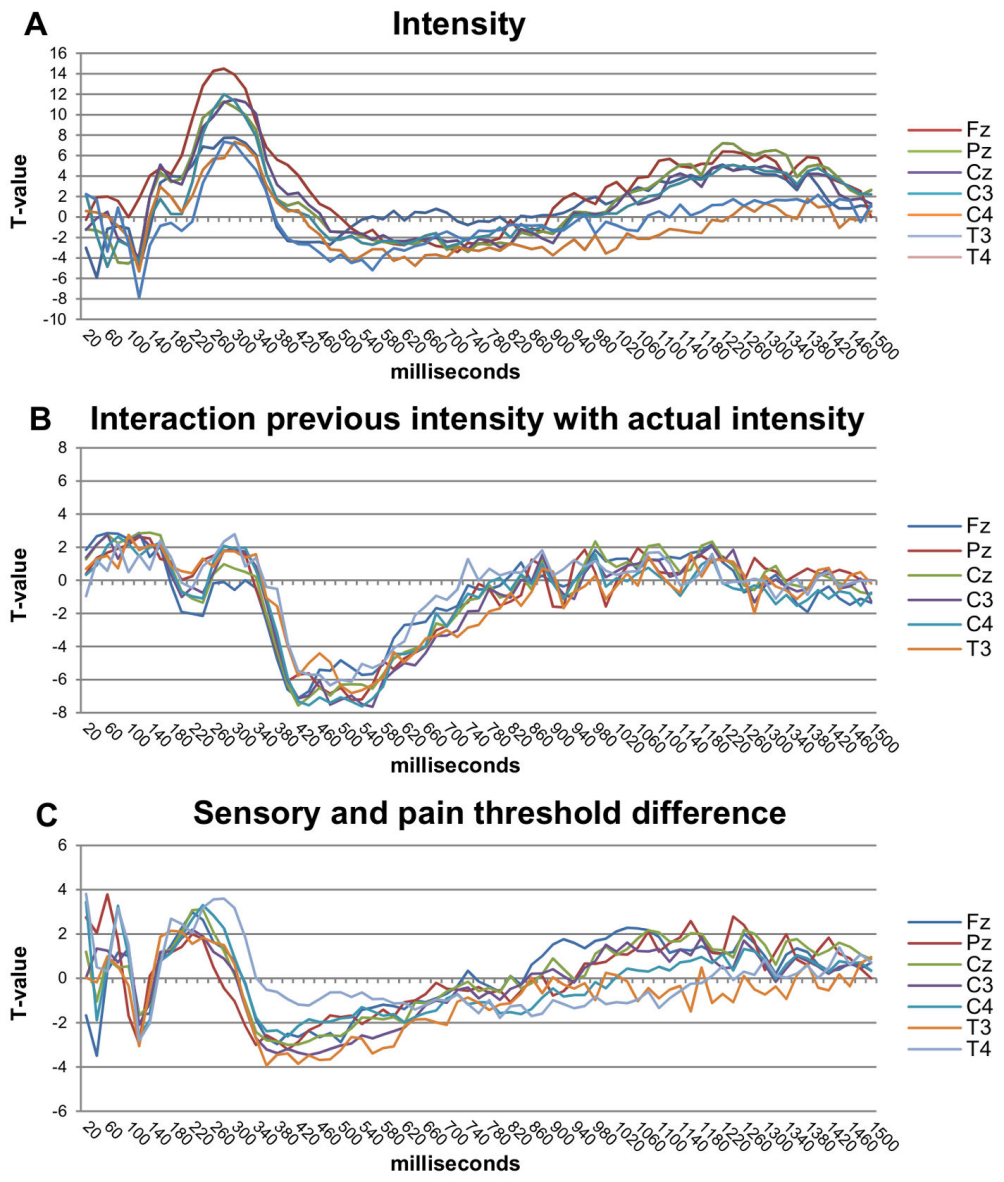
T-value graphs of the stimulus intensity related variables. These graphs show the results of the 2 fixed variables and one interaction of the multilevel model, respectively. A) Stimulus intensity, B) the interaction between the actual stimulus intensity with the previous stimulus intensity, and C) the difference between sensory and pain threshold. On the horizontal axis, the ERFIAs of 75 consecutive 20-ms intervals (0-1500 ms poststimulus) are displayed, with corresponding T-values of the fixed variable from the multilevel analyses on the vertical axis. T-values above 2 or below -2 have a corresponding p-value of 0.05.

**Figure 5:**
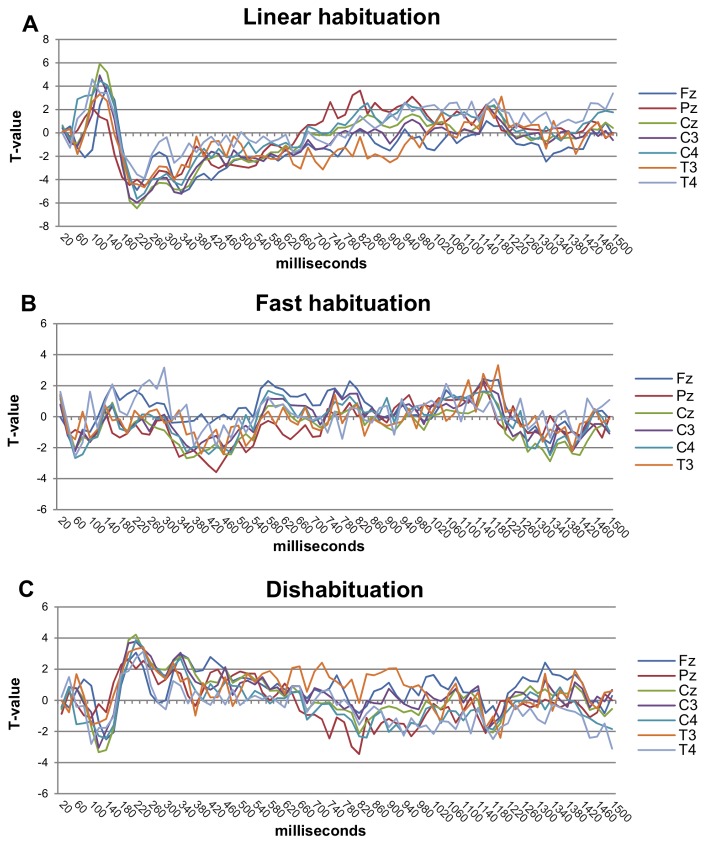
T-value graphs of the habituation variables. These graphs show the results of the 3 habituation variables. A) Linear habituation, B) fast habituation (inverse), and C) dishabituation (quadratic), On the horizontal axis, the ERFIAs of 75 consecutive 20-ms intervals (0-1500 ms poststimulus) are displayed, with corresponding T-values of the fixed variable from the multilevel analyses on the vertical axis. T-values above 2 or below -2 have a corresponding p-value of 0.05.

T-values were plotted for each of the fixed variables of the multilevel models (y-axis) for the 75 consecutive ERFIAs (x-axis) for all 7 EEG electrodes (Figures 4, and 5) to identify significant effects. Figures 4 and 5 show the results for stimulus intensity, linear habituation, fast habituation (trial_inverse_), dishabituation (trial_quadratic_), the difference between the sensory and pain thresholds and the actual stimulus intensity* previous stimulus intensity interaction.

### Stimulus intensity

For stimulus intensity, a significant effect was observed from 100 to 160 ms post-stimulus for all electrodes except Pz (Figure 4a). Negative T-values indicate that stronger intensities are accompanied by larger negative ERFIAs. Further, a robust, long-lasting effect was observed for all electrodes from 220 ms to 360 ms post-stimulus, indicating that stronger intensities result in more positive ERFIAs. In the range of 1120 ms to 1400 ms, a third intensity effect was apparent for all electrodes except T3 and T4.

The variable previous stimulus intensity did not have independent main effects. However, the interaction between actual and previous stimulus intensity was highly significant and persisted from 380 to 660 ms post-stimulus (Figure 4b).

### Habituation: linear, inverse, quadratic

For linear habituation, significant positive T-values developed between 100 and 140 ms, except for Pz. On all electrodes, significant negative T-values were observed between 200 and 360 ms, except for T4. For Fz, Pz, Cz, and C3, the effect was nearly continuously prolonged until 560 ms (Figure 5a). The effect of fast habituation occurred from 340 to 480 ms primarily on Pz and to a lesser extent on Cz and C4 (Figure 5b). The effect of dishabituation was seen on all electrodes predominantly between 200 and 260 ms (Figure 5c).

### Difference between pain and sensory thresholds

Significant effects for the difference between pain and sensory thresholds were observed from 200 to 260 ms for Fz, Cz, C4, and T4. Between 360 and 420 ms, significant negative T-values were visible for all electrodes except T4 (Figure 4c). 

### Random effects

Finally, all random effects (intercepts and random slopes for intensity and trial_linear_) were significant in all models, indicating that both intercepts and slopes varied significantly between subjects.

### Post-hoc analyses

It is generally known that the latency of the P2 peak varies between trials, the so-called latency jitter. This P2 latency variability is thought to be the result of both peripheral, as well as cognitive factors [6,28]. Translating this phenomenon into ERFIAs means that the shift of the latency of a peak on the x-axis is inextricably related to changes in ERFIAs on the y-axis. Thus, it could be hypothesized that the P2-latency variability may confound the intensity and habituation effects on ERFIAs.

To investigate this issue, we computed the latency of the P2 at single trial level with the use of the BrainVision Analyser 2.0, Brain Products, München, Germany. Using the peak export module of the BrainVision Analyser software, the maximum amplitude and corresponding latency were determined for each trial, applying a latency window from 100 to 400 ms. Next, we added the latencies of the P2 for each trial to the original multilevel dataset and incorporated P2-latency as a predictor variable in the multilevel model (see appendix). 

**Figure 6:**
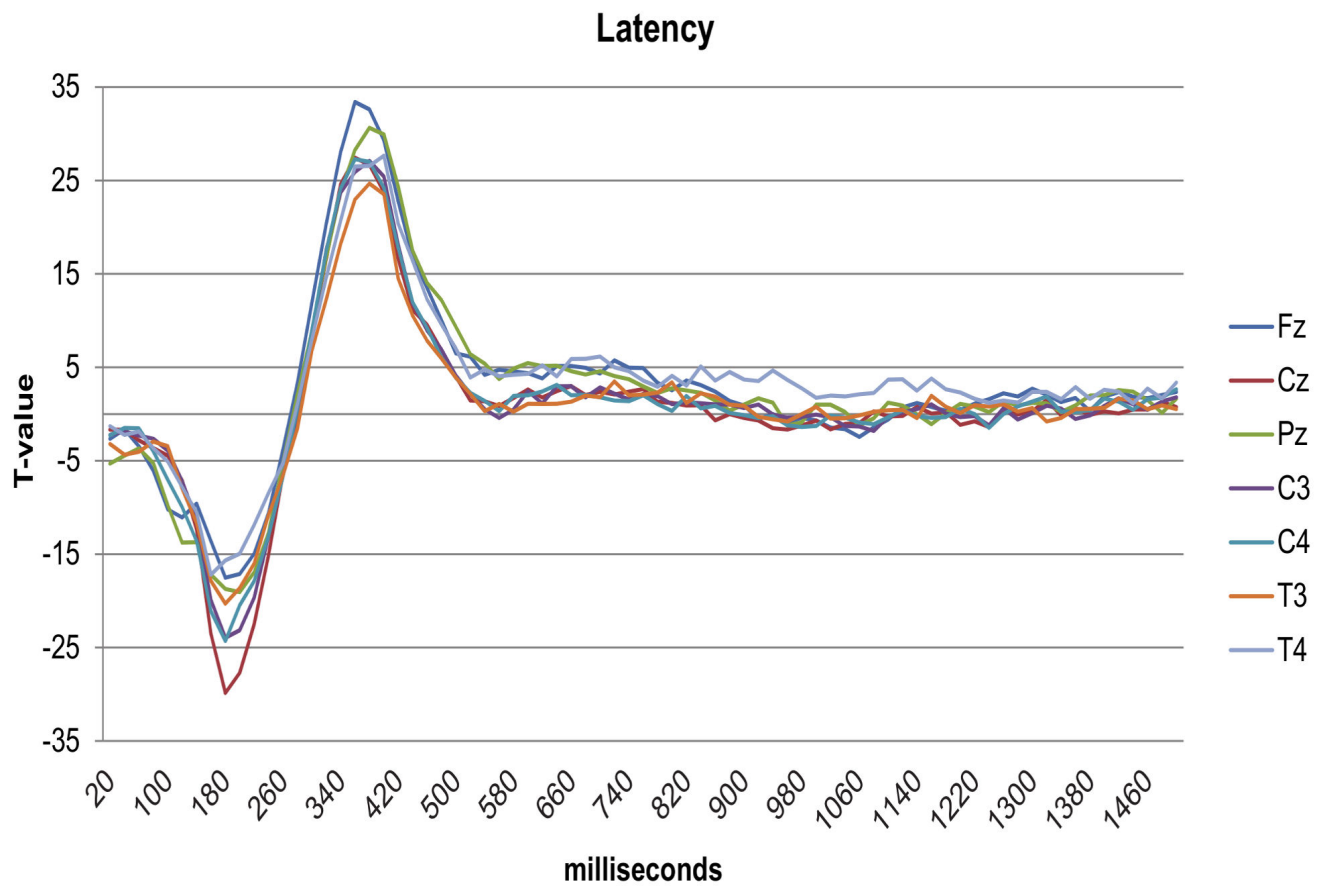
T-value graph of the P2 latency variable. This graph shows the result of the P2 latency variable of the *post-hoc* analyses. On the horizontal axis, the ERFIAs of 75 consecutive 20-ms intervals (0-1500ms poststimulus) are displayed, with corresponding T-values of the fixed variable from the multilevel analyses on the vertical axis. T-values above 2 or below -2 have a corresponding p-value of 0.05.

**Figure 7:**
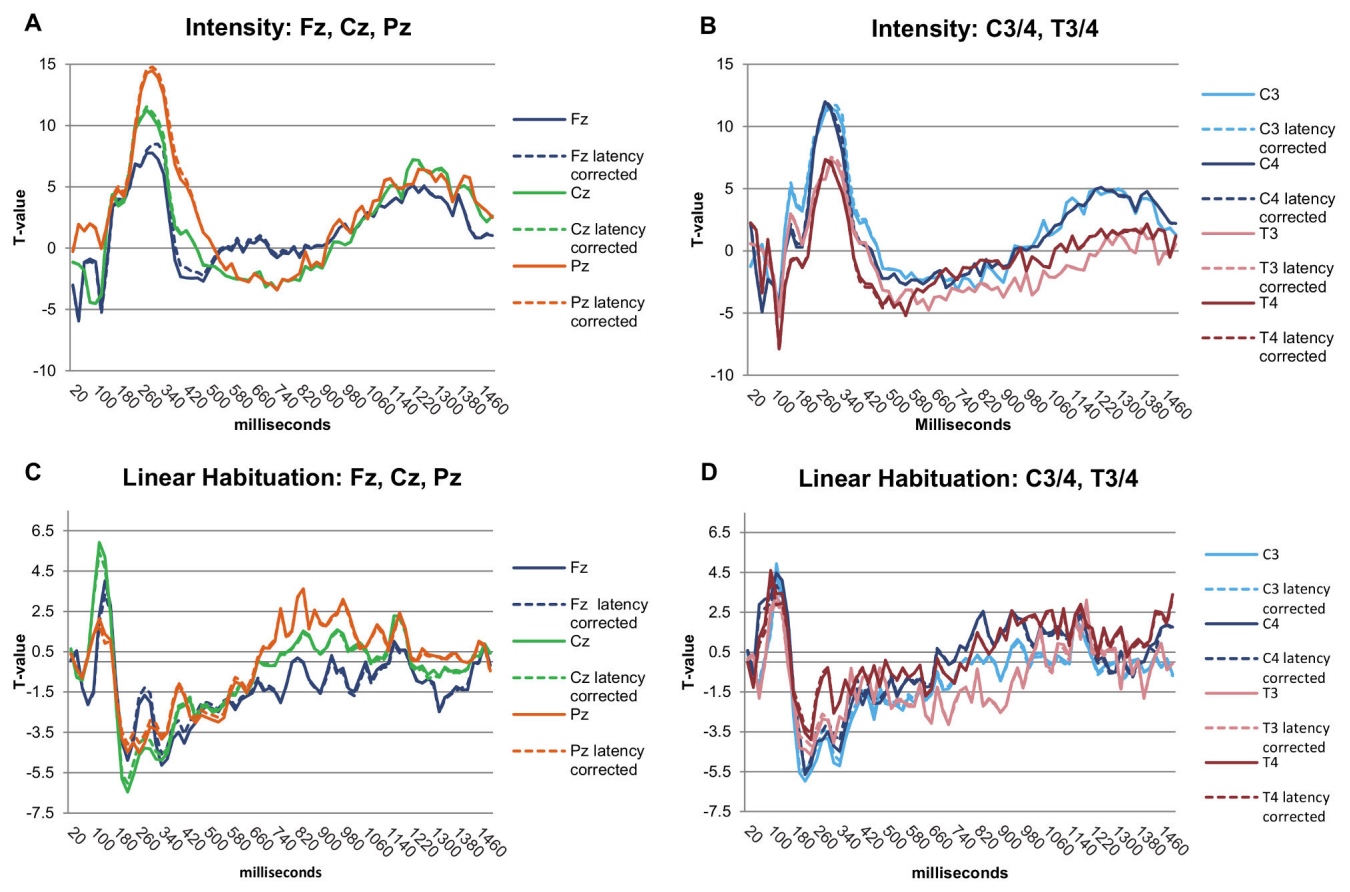
Influence of correction for P2 latency variability on the model. These graphs show the differences between the original model and the model of the *post-hoc* analyses in which the P2 latency variability is incorporated. A) Differences between the models for variable stimulus intensity, at Fz, Cz and Pz. B) Differences between the models for variable stimulus intensity, at C3, C4, T3 and T4. C) Differences between the models for variable linear habituation, at Fz, Cz and Pz. B) Differences between the models for variable linear habituation, at C3, C4, T3 and T4. On the horizontal axis, the ERFIAs of 75 consecutive 20-ms intervals (0-1500 ms poststimulus) are displayed, with corresponding T-values of the fixed variable from the multilevel analyses on the vertical axis.

The results of the main effect of latency variation on ERFIAs are depicted in Figure 6. When examining the T-value curves, we observed a highly significant, sinus shaped effect around the P2. The p-values of the main effects of the other variables remained almost unchanged compared to the original model. For illustrative purposes, the difference in T-value between the models (for intensity and linear habituation) is depicted in Figure 7 for Fz, Cz and Pz. All other electrodes showed similar differences.

## Discussion

In this analysis, the value of a novel analysis of event-related EEG using ERFIAs was examined. This approach was applied to EEG data of a paradigm in which a series of 150 electrical stimuli with 5 intensities was delivered. The first goal was to determine whether the ERFIA multilevel method would yield comparable results with peak analyses of previous studies regarding the effect of stimulus intensity and habituation in the N2 and P2 regions. In addition, we determined the influence of other variables on the complete 1500-ms epoch. 

### Stimulus intensity

Many studies have reported a significant relationship between stimulus intensity and the amplitude of the N2 and P2 peaks. The N2 peak becomes more negative with increasing stimulus intensity, whereas the P2 peak becomes more positive [1,3,6,26,35]. Figure 4a shows that ERFIAs in the N2 and P2 ranges are significantly related to stimulus intensity in the same direction as in the peak analyses. In the ERFIA analysis, the effect of stimulus intensity is not solely expressed in a single peak value but appears to be embedded in a broader range. Further, by ERFIA analysis of the complete 1500-ms epoch, we discovered a strong, long-lasting, ultra-late stimulus intensity effect (from 1000 to 1500 ms) at 5 electrodes. It is unclear what this effect reflects—for example, attention, pain evaluation, or C-fiber activation [36]. Future research is required to clarify this issue. 

### Modeling habituation

In general, habituation is defined as a behavioral response decrement that results from repeated stimulation. The decrease is usually a negative exponential function of the number of stimulus presentations [37,38]. Three forms of habituation can be distinguished overall: an initial rapid decline in response, a linear decrease in response, and an increase in response that reappears after an initial decrement (dishabituation). Habituation to pain can be observed at the level of the subjective pain experience [10] and at the cortical level, expressed in the N2 and P2 peaks of pain-related EEGs [9,13,26]. Like Vossen and colleagues [26,32], we included 3 forms of habituation in the model. 

Regarding linear habituation, significant effects were seen from 100 ms to 160 ms and 180 to 580 ms (Figure 5a). For example, as trial number increased, the ERFIAs became less negative in the N140 range. Similarly, ERFIAs became less positive in the 200-560 range as trial number rose—ie positive ERFIAs returned more quickly to baseline in this range. Notably, intensity and linear habituation influenced ERFIAs oppositely.

The effects of linear habituation were similar to those of the multilevel peak analyses of Vossen, who demonstrated significant effects of linear habituation on the N2 and P2 peaks at all electrodes (Fz, Cz, Pz, C3, C4, T3, T4) [26]. These findings are also consistent with the literature, indicating that habituation is embedded in the N2 and P2 peaks. Valeriani and colleagues [13] studied habituation processes in migraine sufferers and pain-free controls, applying CO2 laser stimulation. In the pain-free control group, habituation on the N2/P2 components was observed. Another study observed that fibromyalgia patients experience reduced habituation on the N2 component[9]. 

In addition to linear habituation, we also found significant effects for both fast (inverse) habituation and (quadratic) dishabituation (Figures 5b and 5c), consistent with earlier findings on peak analyses, but less pronounced compared with the effects of stimulus intensity and linear habituation. Significant effects of linear habituation and dishabituation were noted in the 100-140 ms region, in contrast to fast habituation, implying that the habituation process takes place in disparate parts of the epoch and is a complex function over time. Perhaps other functions may fit the data better than the 3 we used and this could be an interesting topic for future research. 

Overall, the results from this study demonstrate that the influence of stimulus intensity and habituation is not merely limited merely to specific peak values. Both variables appear to exist in a much broader latency range than commonly presumed. The possibility of investigating the effects in late nonpeak-related areas is perhaps the key advantage of the ERFIA technique. Because a deficit in habituation might partially explain chronification of pain, the ERFIA multilevel random regression method can be used to study habituation to stimuli in pain populations compared with pain-free controls. Ideally, longitudinal studies should be performed to examine habituation changes over time in the chronification of pain.

### Post-hoc analyses

Latency jitter is a phenomenon related to peaks. Between trials, the latency of a peak varies. Latency jitter of the P2 may be associated to peripheral as well as cognitive factors [6,28,39]. First, it seems logical that the fixed interval areas under the curve (ERFIAs) are influenced by a change in latency of a peak. Second, it may be hypothesized that the jitter of the P2 confounds the effects of stimulus intensity and habituation on ERFIAs. To test these two critical issues, we incorporated the P2 latency variability in the multilevel model. 

Regarding the main effect of the P2 latency variability, we observed a highly significant, sinus shaped effect around the P2 (Figure 6). This shape makes sense: first, exactly on the averaged P2-peak a zero effect of latency was found. The average peak value corresponds to the mean value of latency. The variable latency exerts a significant negative main effect on ERFIAs before the averaged P2 peak. In other words, in the region *before* the averaged P2 peak, ERFIAs are negatively adjusted when peak latency increases. In the post stimulus region *after* the averaged P2 peak, ERFIAs are positively adjusted with increasing peak latencies. Thus, a shift in the latency of a peak on the x-axis has a profound effect on the y-axis, i.e. ERFIAs. 

Although the effect of the P2 latency variable is highly significant, the T-value plots of all other variables in the model remained almost identical. Thus, despite the large main effects of the variable P2 latency on ERFIAs, there was no confounding effect on the other variables.

In the *post-hoc* analyses, we showed that it is possible to account for latency variations of peaks in the ERFIA multilevel method. In future work, relevant latency jitter of other peaks can also serve as predictor variable. 

### The ERFIA multilevel method: critical evaluation

Although ERFIAs appear to be promising in the study of pain-related somatosensory ERPs, certain critical issues must be addressed. One concern is the optimal width of AUC segments. In this study, we used fixed 20-ms segments to obtain a general impression of effects of the complete 1500-ms post-stimulus range. The width of such a segment should be small enough to obtain sufficient specificity but should not be too small to result in excessive significance testing. When a study focuses on a specific small post-stimulus range, however, it could be argued that this time range should be partitioned into smaller segments, permitting a more detailed examination of the influence of predictor variables. 

Another issue, related directly to AUC width, concerns multiple testing. Because ERFIAs of a specific post-stimulus interval range acts as a dependent variable in a multilevel model, the risk of an unacceptably high number of statistical tests increases as the ERFIA width becomes smaller. In this article, we examined the complete (1500 ms) epoch with 75 20-ms ERFIAs. Thus, as outlined in the methods section, when applying a Bonferroni correction, only P-values that are smaller than 0.0007 are statistically significant. This correction for multiple testing, however, seems to be too rigid. In this report, we proposed the combination of the strict Bonferroni correction for single ERFIAs with a more tolerant, less stringent level of significance for 3 or more consecutive ERFIAs. Future research should examine whether other methods, such as permutation testing and bootstrapping, are more appropriate than the Bonferroni method for correcting multiple testing of ERFIAs.

A third issue relates to the rejection of ERFIAs due to eye movements (EOG). In the analyses, a ±25 μV EOG rejection criterion for each 20-ms ERFIA range was used. After EOG rejection, the minimum amount of ERFIAs that were available in a specific 20-ms range was 8600, which has been deemed sufficient for performing multilevel analyses. It is unclear whether the EOG criterion should be extended to surrounding ERFIAs. For example, in the analysis of an ERFIA at 140 ms, one could consider implementing an EOG rejection for 100-180 ms. Apart from this issue, the use of multilevel analyses is particularly advantageous in handling confounded EOG segments, since all ‘valid’, analyzable segments are included, whereas in the analysis of variance, all observations that pertain to a given subject would be excluded if there are too many invalid segments. It is unknown whether the results are influenced by an adjustment of the EOG rejection criterion.

In the *post-hoc* analyses, we used a straightforward method to determine the latency of the P2 peaks at single trial level, by using a fixed latency window. Of course, other more sophisticated methods for the determination of latencies of peaks, for example as described by Hu and colleagues, can be applied for this purpose [6]. 

### New insights with the ERFIA technique

Notwithstanding the critical issues above, we gained several insights into cortical processing of (painful) electrical stimuli with the use of ERFIA method. Plotting the T-values of a predictor variable against consecutive ERFIAs has an advantage in the interpretation of the results. In the examination of all consecutive ERFIAs of the poststimulus period, predictor variables become more significant, reach a maximum significance level, and subsequently decrease. This pattern gives insight into the approximate onset and end of an influential effect of a variable. For example, intensity appears to have 2 main effects in the latency range of 140-380 ms (Figure 4a), which suggests 2 ‘intensity’ processes.

A novel finding was the prolonged, significant interaction between actual stimulus intensity and previous stimulus intensity, which emerged from 380 to 660 ms on all electrodes. Notably, there was no main effect of previous stimulus intensity, suggesting that previous stimulus intensity is only meaningful in relation to the actual stimulus intensity and that —ie, cortical processing of the actual stimulus intensity is modified considerably by the intensity of the previous stimulus. Thus, the brain may make a “comparison” with previous stimulus intensity information, possibly reflecting stimulus-related memory processes. At approximately 400 ms, this interaction effect was significant, after the main effect of the actual stimulus intensity diminished, suggesting two separate ‘intensity’ processes.

The negative T-values of the interaction can be interpreted as follows: higher previous stimulus intensities result in less positive effect on ERFIAs of the current stimulus intensity, and vice versa—ie, there appears to be a cortical tendency to adjust the actual stimulus intensity in the opposite direction, depending on the level of the previous intensity. As an analogy, one hand is placed in hot water and the other in cold water for a short time, and then both hands are put in warm water; the warm water will feel rather hot to the hand that was immersed in cold water and relatively cold to the other.

More research is required to study the influence of previous stimuli on the processing of the actual stimulus. In addition, greater differences between pain and sensory thresholds or “sensory-pain threshold gap”, result in a greater increase of ERFIAs between 200 and 260ms, and a reduction in ERFIAs between 360 to 420 ms. The clinical implication of this finding and its relation to the experience of pain may be an interesting topic for future research. Given the robust effect on the event-related EEG it seems to be reasonable to include this variable in future analyses. Because the ERFIA multilevel method is a processing technique for raw EEG data, the generalizability of the ERFIA multilevel method seems obvious and can be applied to all types of stimulus-related EEG information. The application of this method to other areas of event-related EEGs and whether the variability of the ERFIA information can be linked to meaningful stimulus- and subject information remains to be determined.

In conclusion, the ERFIA multilevel method enables us to examine the complete post-stimulus period, including non-peak-related information, ultra-late information, and model time-dependent variables, such as habituation, in a refined manner. 

The multilevel ERFIA method will likely contribute to the unraveling of mechanisms of pain-related cortical processing. This method can be applied to all other forms of event-related cortical processing, including other types of noxious stimulation.

## Supporting Information

Appendix S1 **Two multilevel models.** The general model and the post-hoc model corresponding to the analyses of the article “Introducing the Event Related Fixed Interval Area (ERFIA) multilevel technique: a method to analyze the complete epoch of event-related potentials at single trial level”.
(DOCX)

## References

[1] Becker DE, Haley DW, Ureña VM, Yingling CD (2000) Pain measurement with evoked potentials: combination of subjective ratings, randomized intensities, and long interstimulus intervals produces a P300-like confound. Pain 84: 37-47. doi:10.1016/S0304-3959(99)00182-7. PubMed: 10601671. 10601671

[2] Granovsky Y, Granot M, Nir RR, Yarnitsky D (2008) Objective correlate of subjective pain perception by contact heat-evoked potentials. J Pain 9: 53-63. doi:10.1016/j.jpain.2007.08.010. PubMed: 17988951. 17988951

[3] Bromm B, Meier W (1984) The intracutaneous stimulus: a new pain model for algesimetric studies. Methods Find Exp Clin Pharmacol 6: 405-410. PubMed: 6503475. 6503475

[4] Bromm B, Treede RD (1991) Laser-evoked cerebral potentials in the assessment of cutaneous pain sensitivity in normal subjects and patients. Rev Neurol Paris 147: 625-643. PubMed: 1763252. 1763252

[5] Iannetti GD, Zambreanu L, Cruccu G, Tracey I (2005) Operculoinsular cortex encodes pain intensity at the earliest stages of cortical processing as indicated by amplitude of laser-evoked potentials in humans. Neuroscience 131: 199-208. doi:10.1016/j.neuroscience.2004.10.035. PubMed: 15680703. 15680703

[6] Hu L, Liang M, Mouraux A, Wise RG, Hu Y et al. (2011) Taking into account latency, amplitude, and morphology: improved estimation of single-trial ERPs by wavelet filtering and multiple linear regression. J Neurophysiol 106: 3216-3229. doi:10.1152/jn.00220.2011. PubMed: 21880936. 21880936 PMC3234084

[7] Le Pera D, Valeriani M, Niddam D, Chen AC, Arendt-Nielsen L (2002) Contact heat evoked potentials to painful and non-painful stimuli: effect of attention towards stimulus properties. Brain Topogr 15: 115-123. doi:10.1023/A:1021472524739. PubMed: 12537307. 12537307

[8] Miltner W, Johnson R Jr., Braun C, Larbig W (1989) Somatosensory event-related potentials to painful and non-painful stimuli: effects of attention. Pain 38: 303-312. doi:10.1016/0304-3959(89)90217-0. PubMed: 2812841. 2812841

[9] de Tommaso M, Federici A, Santostasi R, Calabrese R, Vecchio E et al. (2011) Laser-evoked potentials habituation in fibromyalgia. J Pain 12: 116-124. doi:10.1016/j.jpain.2010.06.004. PubMed: 20685171. 20685171

[10] Milne RJ, Kay NE, Irwin RJ (1991) Habituation to repeated painful and non-painful cutaneous stimuli: a quantitative psychophysical study. Exp Brain Res 87: 438-444. PubMed: 1769394. 1769394 10.1007/BF00231861

[11] Smith BW, Tooley EM, Montague EQ, Robinson AE, Cosper CJ et al. (2008) Habituation and sensitization to heat and cold pain in women with fibromyalgia and healthy controls. Pain 140: 420-428. doi:10.1016/j.pain.2008.09.018. PubMed: 18947923. 18947923

[12] Valentini E, Torta DM, Mouraux A, Iannetti GD (2011) Dishabituation of laser-evoked EEG responses: dissecting the effect of certain and uncertain changes in stimulus modality. J Cogn Neurosci 23: 2822-2837. doi:10.1162/jocn.2011.21609. PubMed: 21265604. 21265604

[13] Valeriani M, de Tommaso M, Restuccia D, Le Pera D, Guido M et al. (2003) Reduced habituation to experimental pain in migraine patients: a CO(2) laser evoked potential study. Pain 105: 57-64. doi:10.1016/S0304-3959(03)00137-4. PubMed: 14499420. 14499420

[14] Dawson GD (1951) A summation technique for detecting small signals in a large irregular background. J Physiol 115: 2p-3p. PubMed: 14889435. 14889435

[15] Dawson GD (1954) A summation technique for the detection of small evoked potentials. Electroencephalogr Clin Neurophysiol 6: 65-84. doi:10.1016/0013-4694(54)90007-3. PubMed: 13141922. 13141922

[16] Gratton G, Coles MG, Donchin E (1983) A new method for off-line removal of ocular artifact. Electroencephalogr Clin Neurophysiol 55: 468-484. doi:10.1016/0013-4694(83)90135-9. PubMed: 6187540. 6187540

[17] Hoormann J, Falkenstein M, Schwarzenau P, Hohnsbein J (1998) Methods for the quantification and statistical testing of ERP differences across conditions. Behav Res Methods 30: 103-109. doi:10.3758/BF03209420.

[18] Mouraux A, Iannetti GD (2008) Across-trial averaging of event-related EEG responses and beyond. Magn Reson Imaging 26: 1041-1054. doi:10.1016/j.mri.2008.01.011. PubMed: 18479877. 18479877

[19] Woestenburg JC, Verbaten MN, Van Hees HH, Slangen JL (1983) Single trial ERP estimation in the frequency domain using orthogonal polynomial trend analysis (OPTA): estimation of individual habituation. Biol Psychol 17: 173-191. doi:10.1016/0301-0511(83)90018-2. PubMed: 6640015. 6640015

[20] Luck SJ (2005) An introduction to the event-related potential technique. MIT Press.

[21] Mouraux A, Plaghki L (2004) Single-trial detection of human brain responses evoked by laser activation of Adelta-nociceptors using the wavelet transform of EEG epochs. Neurosci Lett 361: 241-244. doi:10.1016/j.neulet.2003.12.110. PubMed: 15135938. 15135938

[22] Quian Quiroga R, Garcia H (2003) Single-trial event-related potentials with wavelet denoising. Clin Neurophysiol 114: 376-390. doi:10.1016/S1388-2457(02)00365-6. PubMed: 12559247. 12559247

[23] Jung TP, Makeig S, Westerfield M, Townsend J, Courchesne E et al. (2001) Analysis and visualization of single-trial event-related potentials. Hum Brain Mapp 14: 166-185. doi:10.1002/hbm.1050. PubMed: 11559961. 11559961 PMC6871967

[24] Hatem SM, Hu L, Ragé M, Gierasimowicz A, Plaghki L et al. (2012) Automated single-trial assessment of laser-evoked potentials as an objective functional diagnostic tool for the nociceptive system. Clin Neurophysiol, 123: 2437–45. PubMed: 22705227. 22705227 10.1016/j.clinph.2012.05.007

[25] Hu L, Mouraux A, Hu Y, Iannetti GD (2010) A novel approach for enhancing the signal-to-noise ratio and detecting automatically event-related potentials (ERPs) in single trials. NeuroImage 50: 99-111. doi:10.1016/j.neuroimage.2009.12.010. PubMed: 20004255. 20004255

[26] Vossen H, Van Breukelen G, Hermens H, Van Os J, Lousberg R (2011) More potential in statistical analyses of event-related potentials: a mixed regression approach. Int J Methods Psychiatr Res 20: e56-e68. PubMed: 21812066. 21812066 10.1002/mpr.348 PMC6878471

[27] Goldstein H (2011) Multilevel Statistical Models. John Wiley & Sons.

[28] Beydoun A, Morrow TJ, Shen JF, Casey KL (1993) Variability of laser-evoked potentials: attention, arousal and lateralized differences. Electroencephalogr Clin Neurophysiol 88: 173-181. doi:10.1016/0168-5597(93)90002-7. PubMed: 7684966. 7684966

[29] Zaslansky R, Sprecher E, Tenke CE, Hemli JA, Yarnitsky D (1996) The P300 in pain evoked potentials. Pain 66: 39-49. doi:10.1016/0304-3959(96)03020-5. PubMed: 8857630. 8857630

[30] Vossen HG, van Os J, Hermens H, Lousberg R (2006) Evidence that trait-anxiety and trait-depression differentially moderate cortical processing of pain. Clin J Pain 22: 725-729. doi:10.1097/01.ajp.0000210913.95664.1a. PubMed: 16988569. 16988569

[31] Luck SJ (2005) Ten simple rules for designing ERP experiments. In: Handy TC Event-related potentials: a methods handbook. MIT Press pp. 17-23.

[32] Vossen CJ, Vossen HGM, Van de Wetering W, Marcus MAE, Van Os J et al. (2012) The use of event-related potentials in chronic back pain patients. In: Norasteh A Low Back Pain In Tech. pp. 1-22.

[33] Klem GH, Luders HO, Jasper HH, Elger C (1999) The ten-twenty electrode system of the International Federation The International Federation of Clinical Neurophysiology. Electroencephalogr Clin Neurophysiol Suppl 52: 3-6. 10590970

[34] Bingel U, Tracey I (2008) Imaging CNS Modulation of Pain in Humans. Physiology 23: 371-380. doi:10.1152/physiol.00024.2008. PubMed: 19074744. 19074744

[35] Stowell H (1977) Cerebral slow waves related to the perception of pain in man. Brain. Res Bull 2: 23-30. doi:10.1016/0361-9230(77)90021-1. 861768

[36] Bromm B, Treede RD (1987) Pain Related Cerebral Potentials: Late and Ultralate Components. Int J Neurosci 33: 15-23. doi:10.3109/00207458708985926. PubMed: 3610490. 3610490

[37] Thompson RF, Spencer WA (1966) Habituation: a model phenomenon for the study of neuronal substrates of behavior. Psychol Rev 73: 16-43. doi:10.1037/h0022681. PubMed: 5324565. 5324565

[38] Rankin CH, Abrams T, Barry RJ, Bhatnagar S, Clayton DF et al. (2009) Habituation revisited: an updated and revised description of the behavioral characteristics of habituation. Neurobiol Learn Mem 92: 135-138. doi:10.1016/j.nlm.2008.09.012. PubMed: 18854219. 18854219 PMC2754195

[39] Mayhew SD, Iannetti GD, Woolrich MW, Wise RG (2006) Automated single-trial measurement of amplitude and latency of laser-evoked potentials (LEPs) using multiple linear regression. Clin Neurophysiol Off J Int Fed Clin Neurophysiol 117: 1331-1344. doi:10.1016/j.clinph.2006.02.017. PubMed: 16644270. 16644270

