# Supplementary material for: Introducing the Event Related Fixed Interval Area (ERFIA) Multilevel Technique: a Method to Analyze the Complete Epoch of Event-Related Potentials at Single Trial Level
Source: PLoS One. 2013 Nov 4;8(11):e79905. doi: 10.1371/journal.pone.0079905 (PMC3817110; doi:10.1371/journal.pone.0079905)
Supplement: Appendix S1 — Two multilevel models. The general model and the post-hoc model corresponding to the analyses of the article “Introducing the Event Related Fixed Interval Area (ERFIA) multilevel technique: a method to analyze the complete epoch of event-related potentials at single trial level”. (DOCX) [file pone.0079905.s001.docx]

**Appendix S1**

The multilevel model:

Y_ti_ = ß_0_ + ß_1_⋅intensity_lin_ + ß_2_⋅trial_lin_ + ß_3_⋅trial_quad_ + ß_4_⋅trial_invers_ + ß_5_⋅age + ß_6_⋅gender + ß_7_⋅difference_pain_and_sensory_threshold + ß_8_⋅intensity_lin__previous_trial +

ß_9_⋅intensity_lin_ ⋅intensity_lin__previous_trial + e_ti_ + u_0i_ + u_1_⋅ intensity_lin_ + u_2_⋅ trial_lin_

The multilevel model for the *post-hoc* analysis:

Y_ti_ = ß_0_ + ß_1_⋅intensity_lin_ + ß_2_⋅trial_lin_ + ß_3_⋅trial_quad_ + ß_4_⋅trial_invers_ + ß_5_⋅age + ß_6_⋅gender + ß_7_⋅difference_pain_and_sensory_threshold + ß_8_⋅intensity_lin__previous_trial +

ß_9_⋅intensity_lin_ ⋅intensity_lin__previous_trial + ß_10_⋅P2 latency + e_ti_ + u_0i_ + u_1_⋅ intensity_lin_ + u_2_⋅ trial_lin_

where:

t = time point (1 to 150)

i = subject

intensity = -2 = -50%, -1 = -25%, 0 = 0%, 1 = 25% and 2 = 50%

trial = 150 trial numbers, centered from –75 to +75

age = centered continuous variable in years

gender = dichotomous variable, -1 = man, 1= woman

difference pain and sensory threshold = absolute pain threshold - absolute sensation threshold

intensity previous trial = -2 = -50%, -1 = -25%, 0 = 0%, 1 = 25% and 2 = 50%

P2 latency = centered continuous variable in ms

e_ti_ = error variance for subject i at time point t*.* This error term is subdivided into a random intercept, a random slope for intensity_lin_, and a random slope for trial_lin_.

The model must be interpreted as follows:

ß_0_ = the outcome mean (amplitude) for the intensity equal to the pain threshold (intensity = 0) at trial number 75 for a male (gender =0) subject with a mean age.

ß_1_ = the mean difference between intensities

ß_2_ = the mean change in linear contrast over the trial

ß_3_ = the mean change in quadratic contrast over the trial

ß_4_ = the mean change in inverse contrast over the trial

ß_5_ = the mean change in amplitude per year

ß_6_ = the mean difference between men and women

ß_7_ = the mean pain and sensory threshold difference

ß_8_ = the relationship between the intensity of the previous trial and the amplitude of the present trial

ß_9_ = the interaction between the effect of the intensity of the previous trial and the intensity of the present trial

ß_10_ = the mean change in latency
